# Stress Responsive bZIP Transcription Factors ATF4 and BACH1 Cooperate With MAF-Family bZIP Protein NRL to Fine-Tune Rod Photoreceptor Gene Expression

**DOI:** 10.1167/iovs.67.6.9

**Published:** 2026-06-05

**Authors:** Kiam Preston, Madhuri Arya, Anjani Kumari, Jacob Nellissery, Matthew J. Brooks, Zachary Batz, Xulong Liang, Gianluca Tosini, Anand Swaroop

**Affiliations:** 1 Neurobiology, Neurodegeneration and Repair Laboratory, National Eye Institute, National Institutes of Health, Bethesda, Maryland, United States; 2 Department of Pharmacology and Toxicology and Neuroscience Institute, Morehouse School of Medicine, Atlanta, Georgia, United States

**Keywords:** gene regulation, protein–protein interaction, retina, stress response, yeast two hybrid

## Abstract

**Purpose:**

Musculoaponeurotic fibrosarcoma (MAF) family basic motif leucine zipper (bZIP) transcription factor neural retina leucine zipper (NRL) determines rod cell fate and controls expression of rod genes in concert with multiple regulatory proteins. Mutations in NRL, its targets, and interacting proteins are associated with retinopathies. Because bZIP heterodimerization expands target sequence selectivity, we set out to identify bZIP protein interactors of NRL.

**Methods:**

Interactors were identified by yeast two-hybrid and co-immunoprecipitation, validated by high-resolution microscopy and proximity ligation, and functionally assessed by reporter assays. We used Cleavage Under Targets and Tagmentation (CUT&Tag) to map activating transcription factor 4 (ATF4) and BTB and CNC homology 1 (BACH1) occupancy and single-cell RNA sequencing (scRNA-seq) to assess gene expression changes in response to their knockdown in mouse retina.

**Results:**

We identified two bZIP proteins, ATF4 and BACH1, as interactors of NRL. We demonstrate a direct interaction of NRL and ATF4 via leucine zipper domain and validate their co-localization in rod photoreceptors. NRL and BACH1 are also partially colocalized, but their interaction likely requires additional factors. Reporter assays show that ATF4 promotes NRL-mediated transactivation of *rhodopsin* promoter, whereas BACH1 appears to act as a suppressor. CUT&Tag revealed shared and distinct binding sites for NRL, ATF4, and BACH1 in promoters of rod-expressed genes, including phototransduction genes. scRNA-seq further indicated a concordance of higher *ATF4* and *NRL* expression with upregulation of phototransduction genes in distinct rod subpopulations.

**Conclusions:**

We suggest that the NRL-mediated gene regulatory network includes transient and stable but context-dependent protein–protein interactions, which control quantitatively precise gene expression patterns in mature rod photoreceptors. Our findings suggest therapeutic potential for retinopathies involving photoreceptor dysfunction through targeted gene expression modulation.

Transcriptional regulatory networks produce unique patterns of gene expression that are necessary to generate cellular and functional diversity.^1^^,^^2^ Complex interactions among transcription factors (TFs) and associated regulatory proteins guide distinct biological processes including development, homeostasis, and disease.^3^^–^^6^ Specialized structural domains, such as homeodomain, basic motif leucine zipper (bZIP), and intrinsically disordered regions (IDRs), in TFs can govern DNA binding and/or interactions with distinct macromolecules.^7^^–^^9^ Both transient and stable interactions allow coordination of signaling pathways in response to extrinsic and intrinsic cellular environments and expand regulatory specificity for gene expression. For example, c-Jun can heterodimerize with Fos to augment and expand gene expression at specific activator protein 1 (AP-1) sites.^10^^–^^13^ Recent technological advances have greatly facilitated the dissection of gene regulatory networks; however, we still have an incomplete understanding of molecular mechanisms that orchestrate precise and dynamic gene expression patterns across functionally diverse neuronal cells.^14^^,^^15^ Equally important is to elucidate how a limited set of TFs can induce unique gene expression patterns, with important implications for cell fate specification and functional maintenance.

Combinatorial interactions among transcription factors and signaling pathways drive the generation of six major classes of neurons from a common pool of progenitor cells during retinal development.^16^^–^^19^ Mammalian photoreceptor cell fate is determined by synergistic and/or antagonistic actions of several TFs, including orthodenticle homeobox 2 (OTX2), achaete-scute homolog 1 (ASCL1), neuronal differentiation 1 (NEUROD1), Onecut, thyroid hormone receptor beta-2 (TRβ2), neural retina leucine zipper (NRL), and cone-rod homeobox (CRX).^20^^–^^22^ CRX transcription factor is critical for photoreceptor differentiation,^23^^–^^25^ with S-cones being the default fate.^20^ NRL, a bZIP protein of the musculoaponeurotic fibrosarcoma (MAF) subfamily of TFs, is an essential determinant of rod photoreceptors,^26^ whereas TRβ2 is needed for M-cone differentiation.^27^ Ectopic expression of NRL in photoreceptor precursors produces rod cells,^28^ and replacement of NRL with TRβ2 leads to M-cones.^29^ Thus, NRL and TRβ2 can generate three distinct outcomes from CRX-expressing early S-cone photoreceptors in concert with additional genetic and epigenetic regulators.^27^^,^^30^

NRL also plays a key role in maintaining rod function throughout life as indicated by its continued high expression and transcriptional control of thousands of rod-expressed genes.^31^^–^^33^ Previous studies have demonstrated modulation of the regulatory activity of NRL by its interaction with CRX and nuclear receptor subfamily 2 group E member 3 (NR2E3).^34^^–^^36^ We have hypothesized that additional NRL binding partners exist, likely interacting via the IDR and leucine zipper domains and enabling precise spatiotemporal regulation in rod photoreceptors. Consistent with this hypothesis, we have recently demonstrated interaction of NRL with several RNA-binding proteins, which are involved in the resolution of DNA–RNA hybrids during transcription or in splicing.^37^ Additionally, NRL targets are shown to be differentially regulated by heterodimerization between NRL and another bZIP protein c-Jun.^38^ Given that homo- and heterodimerization can expand DNA-binding specificity and functional output,^10^^,^^11^ we sought to examine whether NRL might form dimeric complexes with other bZIP proteins.

We here report the identification of two bZIP proteins, activating transcription factor 4 (ATF4) and BTB and CNC homology 1 (BACH1), and validate their interaction with NRL in the retina using co-immunoprecipitation and proximity ligation assays. ATF4 and BACH1 exert synergistic and antagonistic effects, respectively, on NRL-mediated rhodopsin promoter activity in reporter assays. Furthermore, we demonstrate, using Cleavage Under Targets and Tagmentation (CUT&Tag) assays, a significant overlap among potential regulatory elements bound by NRL, ATF4, and BACH1 in the retina and that ATF4 expression correlates with expression of phototransduction genes in distinct rod photoreceptor populations. Thus, our studies provide new insights into NRL-guided transcriptional control of genes in retinal rod photoreceptors and uncover additional opportunities for designing therapies of retinal diseases involving rod dysfunction.

## Methods

### Mouse Strains and Husbandry

All procedures involving mice were approved by the Animal Care and Use Committee of the National Eye Institute (NEI-ASP#650). C57BL/6J mice (WT, *Nrl*-KO, and *Nrl*-gfp) and CD1 mice were kept in a 12-hour light/12-hour dark cycle and fed ad libitum at the National Eye Institute animal facility.

### Antibodies Utilized

See Table 1.

**Table 1.** Antibodies Utilized for Experiments

Antibody	Source	Catalog No.
Rabbit polyclonal anti-ATF4 (1:400)	Santa Cruz Biotechnology	(C-20): sc-200
Rabbit polyclonal anti-ATF4 (1:400)	Proteintech	10835-1-AP
Rabbit polyclonal anti-BACH1(1:100)	Thermo Fisher Scientific	PA5-117013
Rabbit polyclonal anti-H3K27me3 (1:1000)	MilliporeSigma	07-449
Rabbit polyclonal anti-DHX9 (1:200)	Proteintech	17721-1-AP
Goat polyclonal anti-NRL (1:400)	R&D Systems	AF2945
Rabbit anti-NRL (1:1000)	Swaroop Lab^38^	N/A
Donkey anti-rabbit IgG conjugated Alexa Fluor 488 (1:1000)	Thermo Fisher Scientific	A-21206 (RRID: AB_2535792)
Donkey anti-goat IgG conjugated Alexa Fluor 555 (1:1000)	Thermo Fisher Scientific	A-21432 (RRID: AB_2535853)
Donkey anti-goat IgG Alexa Flour 594	Thermo Fisher Scientific	A-11058

### Yeast Two-Hybrid Assays

Yeast two-hybrid (Y2H) assays were performed using a modified Matchmaker System (Clontech Laboratories, Mountain View, CA, USA). For library screening, the prey vector was pGADT7 containing the Gal4 activating domain (630442; Takara Bio USA, San Jose, CA, USA). Two partial NRL domains were synthesized (GENEWIZ, Germantown, MD, USA) to avoid autoactivation driven by full-length NRL. The first construct contained amino acid residues 95 to 175, which included NRL extended homology domain (EHD) with the basic motif (BM). The second construct included amino acid residues 176 to 237 of the bZIP domain. These NRL sequences were subcloned into the bait vector pGBKT7 containing Gal4 binding domain (630443; Takara Bio USA) and then transformed into Y2HGold yeast. The single bait yeast was inoculated at 30°C overnight in 50 mL (−)Trp broth to obtain 7.5 × 10^8^ cells per culture. Yeast cells were pelleted at 3000*g* for 5 minutes, resuspended in 50 mL of (−)Trp broth, and incubated at 30°C for 3 to 4 hours at 225 rpm on a shaker. Yeast cells containing baits were subsequently transformed with 10 µg of human adult retina prey cDNA library prepared in the laboratory. For screening, positive colonies were selected on SD/-Trp/-Leu/X-alpha-gal/Aureobasidin A (DDO/X/A) media to determine β-galactosidase activity and antibiotic resistance as an indicator of a positive interaction with NRL. These interactors were validated by patch plating onto higher stringency SD/-Trp/-Leu/-Ade/-His/X-alpha-gal/Aureobasidin A (QDO/X/A) media. Positive interactor inserts were polymerase chain reaction (PCR) amplified, sequenced using T7 primer, and identified by Blastn and Blastx (National Center for Biotechnology Information, Bethesda, MD, USA). For direct Y2H assays, the procedure was repeated using DNA sequences of the target clone in the pGADT7 vector.

### Cell Culture

Human embryonic kidney 293 (HEK293) cells were cultured in Dulbecco's Modified Eagle Medium (DMEM; 11885084; Thermo Fisher Scientific, Waltham, MA, USA), supplemented with 10% fetal calf serum (S11550; R&D Systems, Minneapolis, MN, USA), as well as 100-units/mL penicillin G and 100-µg/mL streptomycin (15140122; Thermo Fisher Scientific).

### Co-Immunoprecipitation and Western Blotting

The methods for preparation of nuclear extracts from bovine retina or HEK293 cells, as well as for immunoprecipitation (IP), have been described previously.^39^ Protein complexes were eluted from Dynabeads (Thermo Fisher Scientific) by boiling in Laemmli SDS sample buffer (Thermo Fisher Scientific) for 10 minutes at 95°C and loaded with 5 µL of dual color protein ladder (Bio-Rad, Hercules, CA, USA) for gel electrophoresis at 110 V. The proteins were transferred onto polyvinylidene fluoride (PVDF) membranes. Blocking of the PVDF membranes was performed by 1-hour incubation with 5% milk at room before incubation with the primary antibody (1:1000) at 4°C overnight. We then washed the membranes in Tris-buffered saline with 0.1% Tween 20 (TBST) three times for 15 minutes each before incubation for 1 hour in secondary antibody (1:1000) at room temperature. The membranes were washed three times for 15 minutes each with TBST, and SuperSignal West Pico PLUS Chemiluminescent Substrate (34580; Thermo Fisher Scientific) was used to visualize the signal.

### Immunofluorescence Microscopy

The details of immunofluorescence microscopy have been described earlier.^37^ After blocking for 1 hour, paraformaldehyde (PFA)-fixed sections were incubated in primary antibody (rabbit anti-ATF4, 1:100; rabbit anti-BACH1, 1:100; rabbit anti-DExH-box helicase 9 [DHX9], 1:100); and/or goat anti-NRL, 1:400) at 4°C overnight. After washes, sections were incubated in secondary antibody (rabbit Alexa Fluor 488 for DHX9, ATF4, and BACH1; goat Alexa Fluor 555 for NRL) diluted in blocking solution for 1 hour at room temperature. Then, 4′,6-diamidino-2-phenylindole (DAPI; 1:1000) was added to visualize nuclei. Confocal images were acquired on a Leica SP8 two-photon confocal microscope (Leica Microsystems, Wetzlar, Germany). For stimulated emission depletion (STED) imaging, sections were blocked for 3 hours and then incubated overnight at 4°C with primary antibodies (3× the confocal concentration) and DAPI (1:500). Sections were subsequently incubated overnight at 4°C with secondary antibodies diluted in immunocytochemistry buffer: Donkey anti-Goat IgG (H+L) Cross-Adsorbed Secondary Antibody, Alexa Fluor 594 (A-11058; 1:150; Thermo Fisher Scientific) and nanobody anti-rabbit STAR RED (1:100; abberior, Beijing, China). The samples were then mounted with ProLong Glass (P36980; Invitrogen, Carlsbad, CA, USA) and #1.5 coverslips (VWR, 48393-230; Avantor, Radnor, PA, USA). STED images were acquired using a 100× objective on a Leica TCS SP8 STED 3X microscope and deconvolved post-acquisition using Huygens Professional software.

### HEK293 Transfection

Transfections were carried out according to the MilliporeSigma (Burlington, MA, USA) protocol for X-tremeGENE9 transfection reagent. Briefly, we placed DMEM supplemented with 10% fetal bovine serum in a sterile tube and added X-tremeGENE 9 Transfection Reagent (Roche, Basel, Switzerland) and then the plasmids of interest, mixing gently with the pipette. We incubated for 15 minutes at room temperature then added the transformation solution to cells dropwise. After a gentle shake, cells were left to incubate at 37°C for 48 hours before signal detection.

### Proximity Ligation Assay

The proximity ligation assay (PLA) was performed using a Duolink PLA fluorescence kit (MilliporeSigma) per the manufacturer’s instructions. P28 mouse retinal sections or transfected HEK293 cells were postfixed using 4% PFA in PBS for 7.5 minutes at room temperature and washed three times with PBS. Sections were incubated overnight at 4°C in Duolink Blocking Solution (Sigma-Aldrich, St. Louis, MO, USA) for 60 minutes at 37°C along with DHX9 anti-rabbit, BACH1 anti-rabbit, and ATF4 anti-rabbit antibodies and/or goat anti-NRL. After washing, plus and minus PLA probes were diluted 1:5 into Duolink antibody diluent for 1 hour at 37°C. After further washing, the ligation was performed for 30 minutes at 37°C. Following washing, sections were incubated in amplification buffer (diluted 1:5 in deionized water [diH_2_O]) for 100 minutes at 37°C. Then, DAPI at a concentration of 1:1000 was added to the first of the final three washes. Following sufficient washing, slides were mounted with mounting media and coverslipped for imaging with a Leica SP8 two-photon confocal microscope.

### Dual Reporter Assays

Dual reporter assays were performed using a Promega kit (E1960; Promega Corporation, Madison, WI, USA). HEK293 cells were seeded in 24-well plates at a seeding density of 4 × 10^4^/well, and co-transfected with 0.001 µg of cytomegalovirus (CMV)-*Renilla* (0.001 µg), as well as bovine rhodopsin promoter driving firefly luciferase (0.05 µg; 2294bRhop-Luc (Swaroop lab), ref. PMID:24301678) using X-tremeGENE 9 transfection reagent at a ratio of 3:1 and 3 µL of X-tremeGENE 9 transfection reagent to 1 µg of DNA. Additionally, HEK293 cells were co-transfected with 0.25 µg NRL, CRX, ATF4, and/or BACH1 expression constructs in Invitrogen pcDNA4/HisMax A, B, & C Mammalian Expression Vectors (V86420; Thermo Fisher Scientific) containing an N-terminal Xpress tag. Empty pcDNA4c vector was used to adjust the total amount of the transfected DNA to be even among all conditions. Cells were harvested after 48 hours and washed with chilled 1× PBS twice. Cells were then lysed with 100 µL of the passive lysis buffer (Promega Corporation). Firefly and *Renilla* luciferase activities were determined using a dual luciferase reporter system (Promega Corporation) and measured using a GloMax Navigator microplate luminometer (Promega Corporation). *Renilla* luciferase activity was used as an internal control for transfection efficiency. All experiments were repeated three times.

### CUT&Tag Assay

CUT&Tag assays (53165; Active Motif, Carlsbad, CA) were performed according to the manufacturer's instructions. For ATF4, we utilized three independent adult female *Nrlp*-green fluorescent protein (GFP) mouse retinas. GFP flow sorting was performed at the National Heart, Lung, and Blood Institute (NHLBI) cell sorting core facility and resulted in at least 900,000 flow-sorted GFP^+^ rods from each sample. For BACH1, CUT&Tag was performed on whole retinas from three adult female C57BL/6J mice. Samples were then bound to Concanavalin A beads, resuspended in cold antibody buffer containing ATF4 primary antibody (1:75), and incubated overnight at 4°C with orbital rotation. Secondary antibody (1:100) incubation was performed for 1 hour at room temperature with orbital shaking. Cells were washed three times and then treated with pA-Tn5 transposase. Next, tagmentation buffer was added with incubation at 37°C for 1 hour. Tagmentation was stopped by adding buffer containing 4.2 µL 0.5-M ethylenediaminetetraacetic acid (EDTA), 1.25 µL of 10% sodium dodecyl sulfate (SDS), and 1.1 µL Proteinase K (10 mg/mL) for 1 hour at 55°C. Finally, DNA was purified, amplified by PCR, indexed, and sequenced on a NextSeq 1000/2000 sequencing system (Illumina, San Diego, CA, USA).

### CUT&Tag Analysis

CUT&Tag data were analyzed through the Nextflow 24.04.4/nf-core/cutandrun 3.1 pipeline. Briefly, sequences were paired-end trimmed by Trimgalore 0.6.6 for library adapters (CTGTCTCTTATA). Alignment to *Mus* *musculus* GRCm38 assembly was performed by Bowtie2 2.4.4. Duplicate reads were removed using Picard 2.27.4-SNAPSHOT, BigWig files were generated, and peaks were called using SEACR 1.3. Consensus peaks and overlap with gene promoters were identified using bedtools multiinter and intersect tools, respectively. Read counts per accessible assay for transposase-accessible chromatin (ATAC) footprint were calculated with bedtools coverage. Motif enrichment was performed using the findMotifsGenome feature in Homer. Enrichment analyses of genes bound by multiple TFs were performed using clusterProfiler 4.10.0 and ReactomePA 1.46.0 then plotted using enrichPlot 1.22.0. Genomic region maps and peak coverage heatmaps were generated with ChIPseeker 1.42.1.

### Short-Hairpin RNA Subretinal Injection and Electroporation

Short-hairpin RNA (shRNA) cocktails were made by mixing each shRNA for the gene of interest in equal part with PBS. Electroporation was carried out in 8 to 12 newborn CD-1 mice (Charles River Laboratories, Wilmington, MA, USA) at postnatal day 2 (P2), as described previously.^41^ Briefly, the shRNA was cloned into the AgeI and EcoRI sites using the pLKO.1-puro vector. The shRNA cocktail (0.4 µL at 2.5 µg/µL) and *Nrlp*–enhanced green fluorescent protein (eGFP) vector (1.5 µg/µL, 0.4 µL) were injected subretinally into pups anesthetized on ice. Then, five 80-volt, 50-ms pulses (0.10–0.20 mA) were applied at 950-ms intervals via PBS-soaked tweezer electrodes to electroporate the shRNA and *Nrlp*-eGFP into the retina.

### RNA Extraction and Single-Cell RNA Sequencing

GFP^+^ regions of electroporated retina from 8 to 12 CD-1 mice at P21 to P23 were collected and dissociated as previously described.^40^ Single-cell suspensions of GFP^+^ regions were used as input for Chromium Controller 3.1 (10x Genomics, Pleasanton, CA, USA). Approximately 16,500 cells per sample were loaded into the chromium chip using a cell suspension volume calculator table to target 10,000 cells for recovery. The library was prepared for sequencing according to the manufacturer's instructions (Chromium Single Cell 3′ protocol; 10x Genomics) and sequenced on the NextSeq 1000/2000 platform.

### Single-Cell RNA Sequencing Analysis

Demuliplexing, barcode processing, gene counting, and aggregation were performed with Cell Ranger 8.0.0 with the –force-cells 10k option using a modified mm10 reference. We chose to use the force-cells option because automated cell calling in Cell Ranger was generating many more cells than the predicted cell recovery. To align GFP reads from the control vector *Nrlp*–eGFP^42^ in our shRNA samples, we constructed a modified reference of the 10x Genomics mm10 reference (refdata-gex-mm10-2020-A) containing the GFP sequence from pEGFP-1 plasmid. Data matrixes were imported into Seurat 4.3.0^43^ in the R 4.2.3 statistical environment (R Foundation for Statistical Computing, Vienna, Austria). Initial matrix filtering per sample of >5 cells per gene, >500 features per cell, and cells with <10% mitochondrial reads was performed to ensure that high-quality cells were kept for analysis. Ambient RNA removal was required and performed using the decontX function in the celda 1.14.2 package. DecontX-adjusted count matrixes from all samples were merged, and normalization and variance stabilization were performed using SCTransfrom 2. PCA (50 dimensions), FindNeighbors (30 dimensions), RunUMAP (30 dimensions), and FindClusters (original Louvain algorithm, res = 0.8) were used with Seurat. Cell types were assigned using scType (https://github.com/IanevskiAleksandr/sc-type) with the sctype “eye” database, “Astrocytes,” “Extracellular matrix cells,” “Retinal pigment epithelial cells,” “Fibroblasts,” and custom cell type gene lists (https://github.com/MBrooks313/scRNA-seq/blob/main/annotation/Retina_Cell_type_MB_v4.xlsx). Cluster 16 was removed from the final analysis because of conflicting positive markers for rods and Müller glia, likely representing doublet cells.

The Wilcoxon rank-sum test in the Seurat function FindMarkers was used to determine differential gene expression (DGE). For each comparison, the data were subset for a particular cluster being investigated. DGE was performed using log-normalized decontX-adjusted counts with the following settings in FindMarkers: min.pct = 0.25 and logfc.threshold = log(1.5). Additionally, DGE gene lists were filtered for those having an adjusted *P* < 0.01. Functional gene enrichment of DGE results was performed using clusterProfiler 4.6.2 with the Gene Ontology (GO) biological process database from org.Mm.eg.db 3.16.0 (GOSOURCEDATE: 2022-07-01) as the pathway reference. The results were filtered for pathways having an adjusted *P* < 0.05. To reduce the redundancy of pathways inherent in GO analysis results, pathways were filtered for the most child term of any parent–child term passing significance.

## Results

### NRL Interacts With ATF4 and BACH1

**Figure 1.**
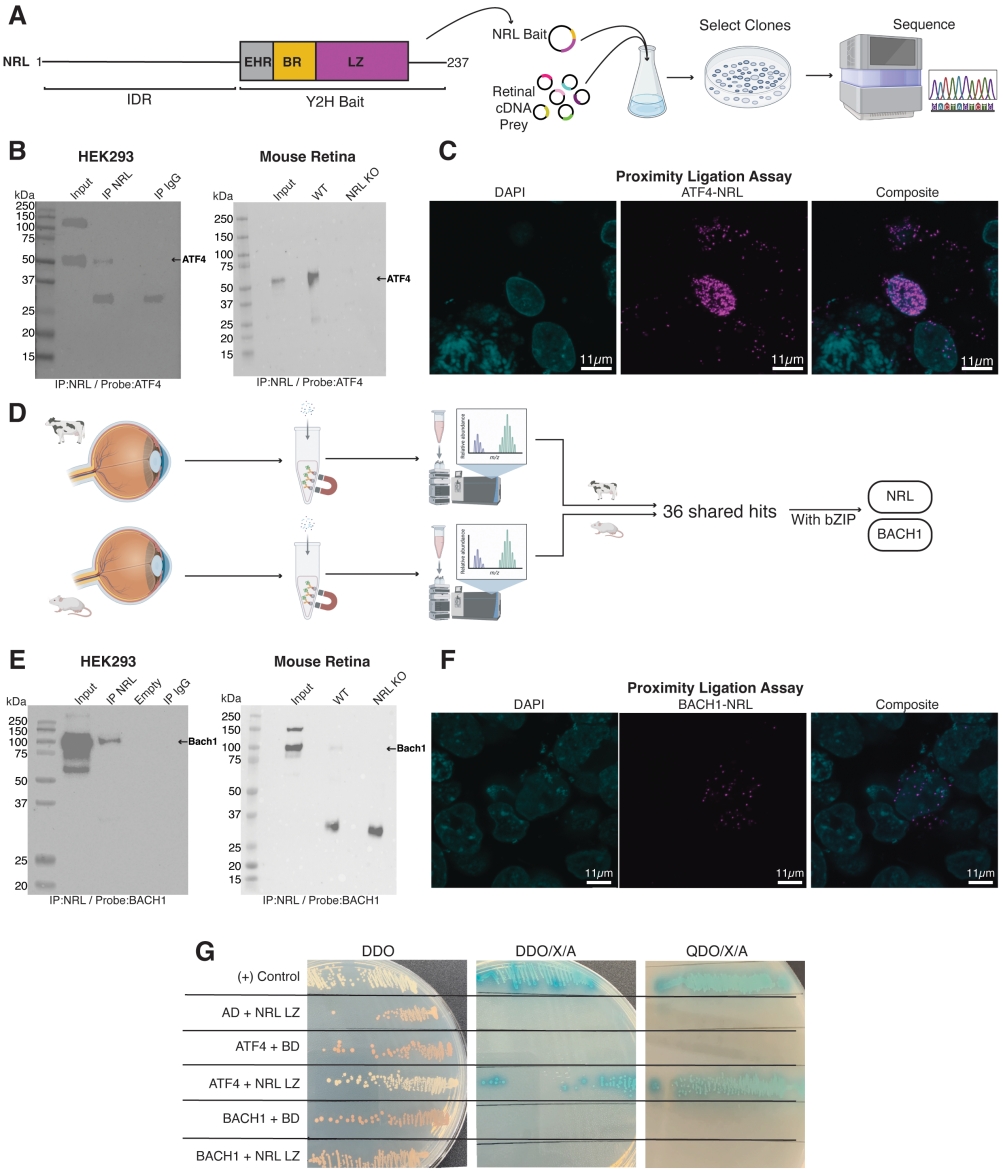
**Identification of candidate NRL interacting proteins ATF4 and BACH1.** (**A**) NRL domains used for Y2H assays and overview of the screening approach. (**B**) Co-immunoprecipitation from nuclear extracts of NRL followed by western blot using ATF4 as the probe in HEK293 cells (*left*) and murine retina (*right*). (**C**) PLA for ATF4 and NRL in HEK293 cells. *Teal* indicates DAPI nuclear stain; *magenta*, PLA signal for ATF4-NRL interaction. (**D**) Co-immunoprecipitation approach used for the identification of BACH1 from bovine (cow) and murine (mouse).^37^ (**E**) Co-immunoprecipitation from nuclear extract of NRL followed by western blot using BACH1 as the probe in HEK293 cells (*left*) and murine retina (*right*). (**F**) PLA for BACH1 and NRL in HEK293 cells. *Teal* indicates DAPI nuclear stain; *magenta*, PLA signal for BACH1–NRL interaction. (**G**) Y2H screening performed on DDO, and DDO/X/O, QDO/X/A plates. AD, pGADT7 empty vector; BD, GBKT7 empty vector; BR, basic region; DDO, double dropout agar; DDO/X/O, double dropout agar with X-Alpha-Gal and Aureobasidin A; IDR, intrinsically disordered domain; IgG, immunoglobulin G; LZ, leucine zipper; QDO/X/A, quadruple dropout agar with X-Alpha-Gal and Aureobasidin A.

To identify NRL interactors, we first performed three rounds of Y2H a assays using retinal cDNA libraries with the leucine zipper domain of NRL as bait. We identified >50 candidate interactors (Fig. 1A); of these, the most frequently detected protein was ATF4 (Table 2, which shows the eight most robust candidates). To validate their direct interaction, we transfected HEK293 cells with an NRL–Flag vector. IP with the anti-Flag antibody (see Table 1 for a list of antibodies) was able to pull down transfected NRL (Supplementary Fig. S1A) and endogenously expressed ATF4 protein (Fig. 1B, left panel). We then performed PLAs in vitro using HEK293 cells transfected with an NRL expression construct. A robust nuclear signal was evident for ATF4-NRL (Fig. 1C), as well as for the reported NRL interactor DHX9,^37^ in the PLAs (Supplementary Fig. S2A), but not for the empty vector negative control (Supplementary Fig. S2B). Co-immunoprecipitation experiments using the wild-type (WT) and *Nrl^–/–^* (*Nrl*-KO) retina lysates showed that anti-NRL antibody can immunoprecipitate ATF4 in WT but not in *Nrl*-KO mice, as predicted (Fig. 1B, right panel), further corroborating in vivo interaction of NRL and ATF4.

As a complementary approach, we utilized existing and previously reported retinal lysate co-IP followed by mass spectrometry data from cow and mouse.^38^^,^^39^ We identified 36 common proteins between the two independent NRL IP (pulldown) experiments and discovered only one bZIP protein, BACH1, in addition to NRL (Fig. 1D). We validated the NRL–BACH1 interaction in NRL–Flag transfected HEK293 cells using both co-immunoprecipitation (Fig. 1E, left panel) and PLA (Fig. 1F). Finally, we confirmed that anti-NRL antibody could immunoprecipitate BACH1 from WT mouse retinal lysate but not from *Nrl*-KO retina lysate (Fig. 1E, right panel). In reverse co-immunoprecipitation experiments, we were unable to immunoprecipitate NRL from WT mouse retina nuclear extracts by either ATF4 or BACH1 antibody, likely due to context-dependent (or transient) interaction of NRL with ATF4 and BACH1, unavailability of respective epitopes, or, in the case of BACH1, its low expression in rods.

Next, we investigated whether the leucine zipper domain of NRL is directly involved in facilitating the interaction with ATF4 and/or BACH1 by performing Y2H assays. Transformation of yeast with the leucine zipper domain of NRL as bait and ATF4 as prey resulted in strongly blue colonies that survive on Aureobasidin A–containing plates; however, no yeast colonies were detected on these plates with BACH1 as prey (Fig. 1G; Supplementary Fig. S1B). Together, these data indicate a direct interaction of NRL with ATF4 potentially via dimerization of their leucine zipper domains, whereas NRL and BACH1 interaction requires additional proteins.

### Interaction of NRL With ATF4 and BACH1 in Photoreceptors

**Figure 2.**
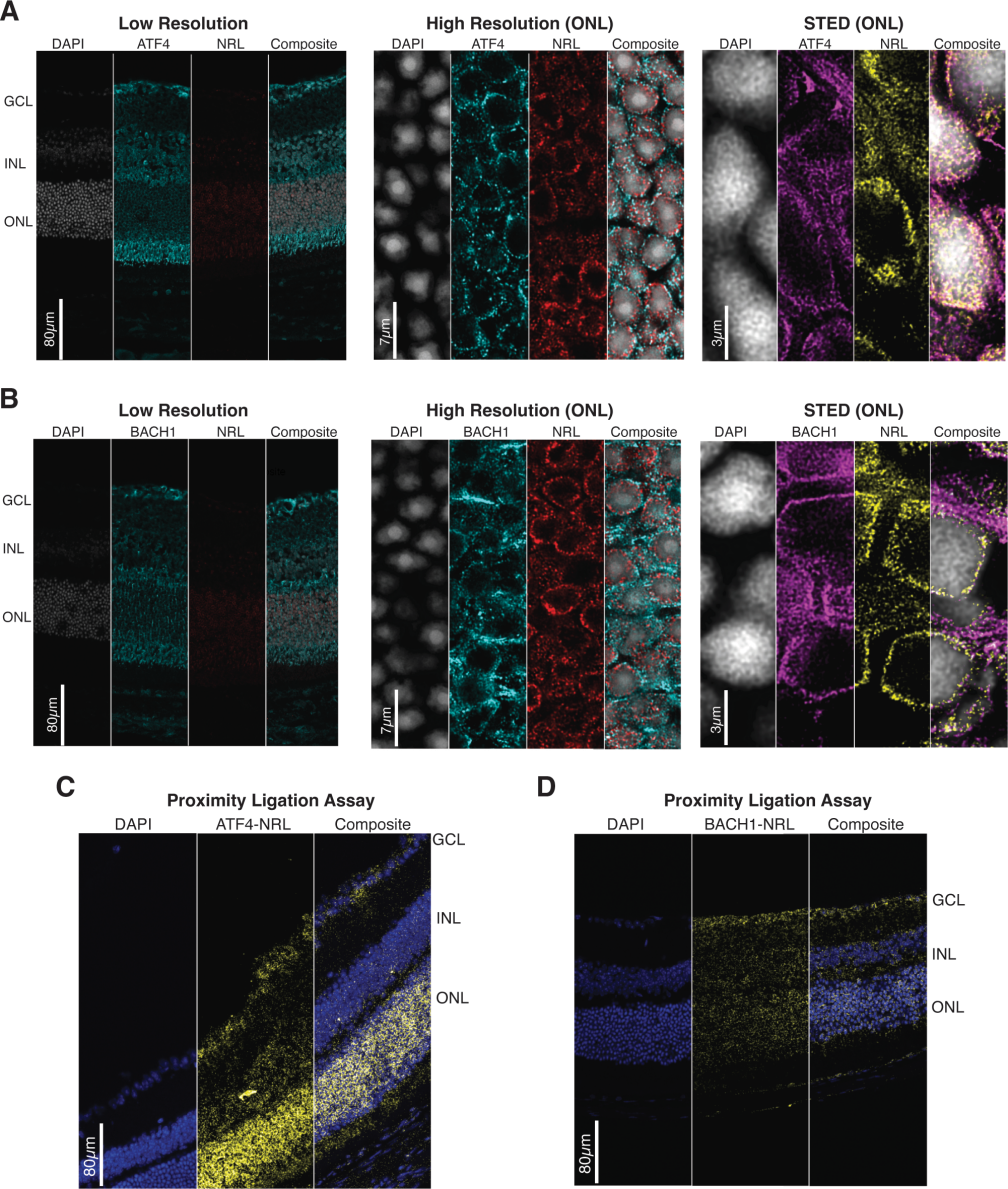
**NRL localizes with ATF4 and BACH1 in rod photoreceptor nuclei.** (**A**) Immunofluorescent staining of ATF4 and NRL in mouse retina. *Gray* indicates DAPI; *teal/magenta*, ATF4; *red/yellow*, NRL. (**B**) Immunofluorescent staining of BACH1 and NRL in mouse retina. *Gray* indicates DAPI; teal/magenta, BACH1; *red/yellow*, NRL. (**C**, **D**) PLA on mouse retina sections for ATF4 and NRL or BACH1 and NRL, respectively. *Blue* indicates DAPI; *yellow*, PLA signal. INL, inner nuclear layer; GCL, ganglion cell layer; ONL, outer nuclear layer; STED, stimulated emission depletion microscopy.

To examine the expression of ATF4 and BACH1 in photoreceptors, immunofluorescence imaging was performed using WT mouse retinal sections. No primary antibody controls showed any signal throughout the WT retina (Supplementary Fig. S2C), whereas, as reported previously,^37^ NRL and DHX9 exhibited uniquely perinuclear staining of euchromatin in rod photoreceptors (Supplementary Fig. S2D). ATF4 colocalizes with NRL in the rod perinuclear region (Fig. 2A). BACH1 displays a similar nuclear staining in rods but with additional signal detectable in the cytosol (Fig. 2B).

To visualize in vivo interactions, we implemented PLA using WT mouse retina sections. A robust PLA signal was evident in WT retinal rods for ATF4–NRL pairs (Fig. 2C) and a similar signal but of lower intensity for BACH1–NRL (Fig. 2D). DHX9–NRL interaction was used as a positive control for PLA in WT retina (Supplementary Fig. S2E). Low-level background was detected in DHX9–NRL PLA using the *Nrl*-KO retina (Supplementary Fig. S2F). Very low-level background was detected when using secondary antibody control with both the WT and *Nrl*-KO retina (Supplementary Figs. S2G, S2H). Similar low-level background was also evident in PLAs for ATF4–NRL (Supplementary Fig. S2I) and BACH1–NRL (Supplementary Fig. S2J) using the *Nrl*-KO retina. These results demonstrate expression of ATF4 and BACH1 together with NRL and their interaction in euchromatin of rod photoreceptor nuclei.

### ATF4 and BACH1 Differentially Modulate NRL-Mediated Rhodopsin Promoter Activity

**Figure 3.**
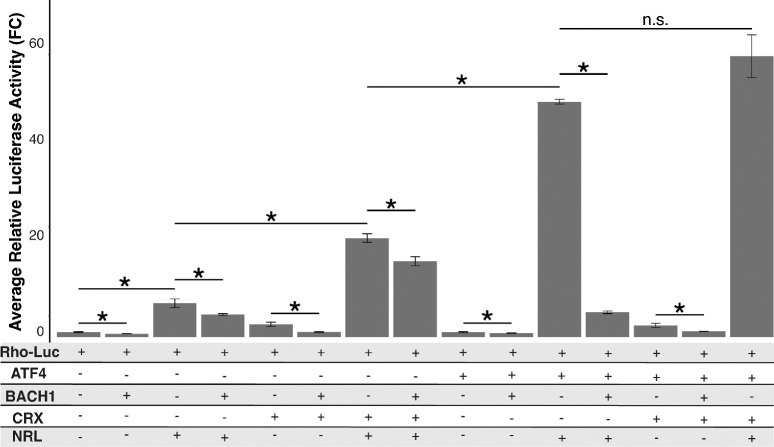
**ATF4 and BACH1 modulate NRL-mediated rhodopsin promoter activity.** NRL-mediated *bRho* promoter (2.2 kb) activity driving luciferase expression in HEK293 cells. Fold change (FC) was calculated relative to activity of the *bRho* promoter alone driving luciferase expression. Presence (+) or absence (−) of transfected plasmids is indicated below each bar. Significant differences were statistically determined by Kruskal–Wallis test followed by pairwise-Wilcoxon test using the Benjamini–Hochberg procedure to control for false discovery rate. *P < 0.05; n.s., not significant. Rho-Luc, 2.2-kb rhodopsin–luciferase construct.

Rhodopsin promoter activity assays in cultured cells are widely used to evaluate transcriptional regulatory activity of WT and mutant NRL, as well as their interaction with CRX and other proteins.^44^^–^^46^ We performed dual reporter assays in HEK293 cells using 2.2-kb *Rho* promoter driving luciferase to investigate functional relationships of ATF4 and BACH1 with NRL and CRX (Fig. 3). As previously established, NRL and CRX can individually (6.9- and 2.6-fold, respectively) and synergistically (20-fold) enhance luciferase reporter expression that is driven by the *Rho* promoter.^34^ ATF4 augments luciferase expression synergistically with NRL (48-fold) but does not influence rhodopsin promoter activity on its own. Additional increase in reporter activity is not statistically significant when CRX is also included with NRL+ATF4. In contrast to ATF4, BACH1 represses the luciferase activity. Notably, BACH1 addition resulted in significantly lower luciferase expression than that induced by NRL, NRL+ATF4, CRX, or NRL+CRX (Fig. 3). These results suggest that ATF4 is a synergistic activator of rod gene expression with NRL, whereas BACH1 functions as a repressor (or negative modulator) of NRL-mediated transcription.

### ATF4 and BACH1 Bind Phototransduction Gene Promotors In Vivo

We performed CUT&Tag using anti-ATF4 and anti-BACH1 antibodies to identify retinal gene promoter regions that ATF4 and BACH1 occupy and compared these to previously reported NRL binding data.^38^ We noted an enrichment of ATF4 and BACH1 peaks at transcription start sites (Supplementary Fig. S3A). Furthermore, a majority of these ATF4 (63.4%) and BACH1 (77.0%) peaks are identified in accessible loci based on existing retina ATAC-sequencing data (Supplementary Fig. S3B).^47^ Conversely, just 9.0% of the ATAC footprints contained BACH1 and/or ATF4 peaks (Supplementary Fig. S3C), and a majority of the accessible regions did not have significant CUT&Tag reads (Supplementary Fig. S3D). The BACH1 motif was enriched among BACH1 peaks relative to both the whole genome (+1.15 log_2_ enrichment; *P* = 1 × 10^–42^) and ATAC peaks (+3.52 log_2_ enrichment; *P* = 1 × 10^–^^245^). Likewise, the ATF4 motifs were enriched among the ATF4 peaks relative to the genome (+0.77 log_2_ enrichment; *P* = 1 × 10^–^^5^) and ATAC peaks (+0.55 log_2_ enrichment; *P* = 0.01), although to a lesser degree than observed for BACH1.

**Figure 4.**
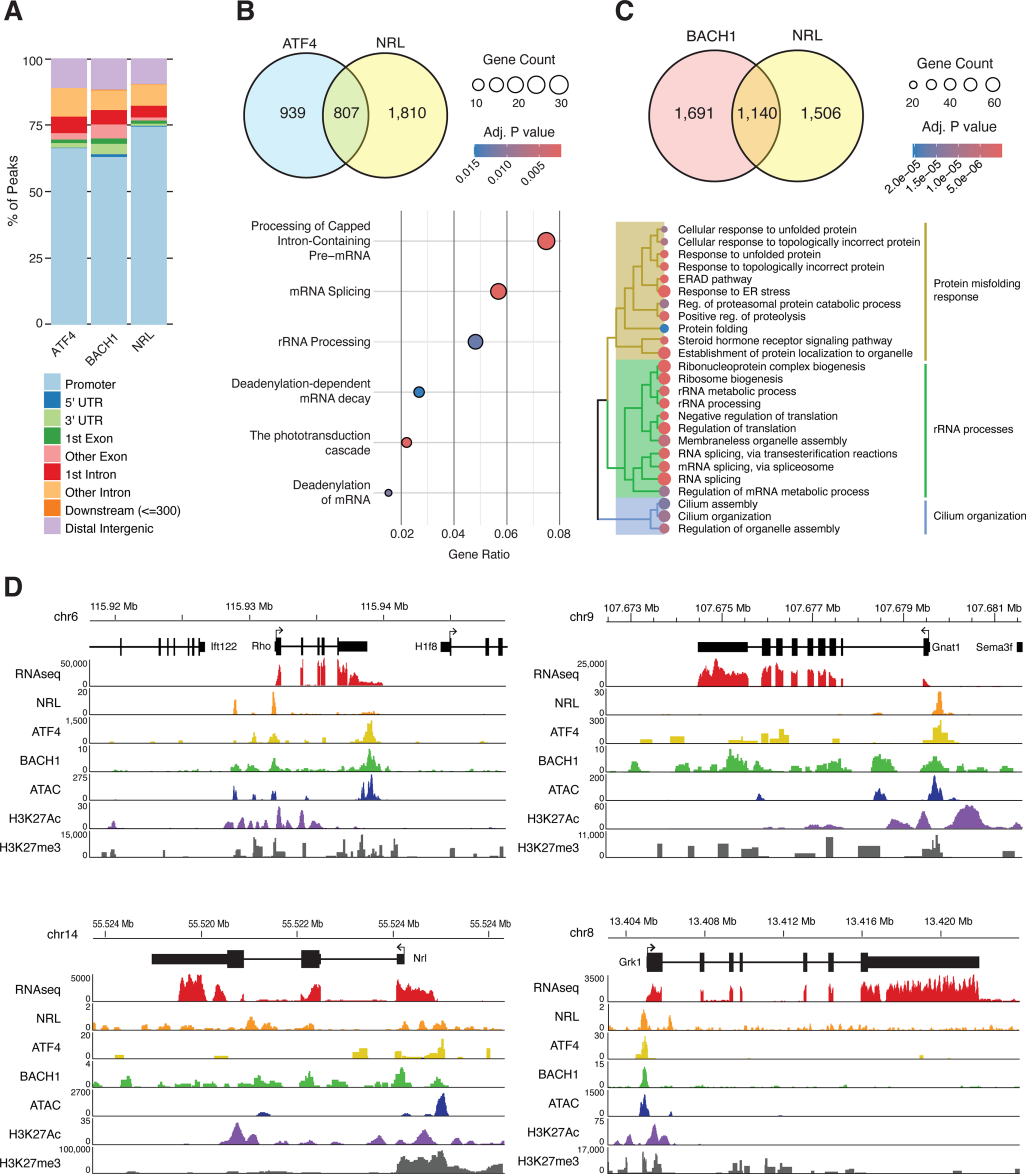
**ATF4 and BACH1 interact with NRL at gene promoters and modulate expression.** (**A**) Distribution of identified peaks across genomic features for ATF4, BACH1, and NRL. (**B**) Shown are the number of protein coding genes having promoter peaks overlapping between ATF4 and NRL (*upper*) and dot plot of Reactome terms enriched for genes with co-bound promoters (*lower*). *Dot size* and *color* indicate the number of co-bound genes on the pathway and the adjusted *P* values, respectively. (**C**) Number of protein coding genes having promoter peaks overlapping between BACH1 and NRL (*upper*) and tree plot of Reactome terms enriched for genes with co-bound promoters (*lower*). Enriched terms are clustered by similarity using pairwise_termsim from enrichplot. *Dot size* and *color* indicate the number of co-bound genes on the pathway and adjusted *P* values, respectively. (**D**) Genomic histogram traces of gene expression, transcription factor binding (NRL, ATF4, and BACH1), genome accessibility (ATAC-seq), and active and repressive histone marks (H3K27ac and H3K27me3) near the phototransduction genes, *Rhodopsin*, *Gnat1*, *Nrl*, and *Grk1*. Adj. *P* value, Benjamini–Hochberg adjusted *P* value; UTR, 5′ untranslated region.

Feature distribution analyses revealed that 70% to 75% of NRL and ATF4 peaks were detected in regions identified as promoters compared to 27% for BACH1 (Fig. 4A); 40.3% of BACH1-bound gene promoters and 46.2% of ATF4-bound gene promoters also exhibited binding of NRL. The 807 gene promoters occupied by both ATF4 and NRL (Supplementary Table S1) were enriched for phototransduction genes (Fig. 4B; Supplementary Table S2). On the other hand, 1140 gene regions bound by both BACH1 and NRL (Supplementary Table S1) included genes associated with common cellular processes (Fig. 4C; Supplementary Table S3), such as RNA processing, which have previously been linked to NRL regulation.^37^

We also noticed that NRL, ATF4, and BACH1 were bound to the promoter regions of many phototransduction genes, including *Rhodopsin*, *Gnat1*, *Nrl*, and *Grk1* (Fig. 4D), indicating their potential role in finetuning expression of rod-specific genes. Other functionally notable gene promoters include ATF4 targets *DDIT3/CHOP* and *Gadd45a*, BACH1 target *Hmox1*, and circadian genes such as *Cry1*, *Per2*, and *Nr1d1* (Supplementary Table S1).

### ATF4 Expression Correlates With Phototransduction Activity and Rod Photoreceptor Specialization

We then performed single-cell RNA sequencing (scRNA-seq) of mouse retina electroporated in vivo with *Atf4* or *Bach1* shRNA (Table 3), which upon transfection into mouse embryonic fibroblasts showed a reduction in the respective protein by immunoblot analysis (Supplementary Figs. S4A, S4B). Single-cell transcriptomic profiles were obtained from *Atf4* shRNA knockdown (KD), *Bach1* shRNA KD, control injected (CNTL_Inj), and non-injected (NON_Inj) whole retina, and total unique molecular identifier (UMI) counts used in further analyses. After filtering, we had 39,579 high-quality cells with a median of ∼2300 counts per cell and ∼1300 genes per cell (Supplementary Figs. S5A–S5F). To facilitate retrieval of *Atf4* and *Bach1* KD cells, all subretinal injections contained a plasmid expressing GFP under the control of the *Nrl* promoter. Although we observed positive GFP expression by microscopy, only a few GFP sequence reads were captured in the scRNA-seq analysis. Without robust GFP identification, we are unable to directly identify cells with *Atf4* or *Bach1* KD at the RNA level. Nonetheless, violin plot density distributions of *Atf4* expression in Atf4^+^ rods suggest that the *Atf4*-KD samples exhibited a trend of reduced *Atf4* expression across most clusters compared to controls (Supplementary Fig. S5F, left panel). Because of low *Bach1* expression across all sample types, further analysis was not pursued (Supplementary Fig. S5F, right panel).

**Table 2.** Appended List of Genes Identified Across Three Independent Yeast Two-Hybrid Assays

	Positive Colonies		
Gene	Replicate 1	Replicate 2	Replicate 3
*ATF4*	4	17	3
*SAG*	2	—	2
*CLUL1*	2	1	1
*ARR3*	1	1	—
*PCNA*	2	—	—
*VIM*	—	2	—
*ASHA1*	2	—	—
*OVOS2*	2	—	—

Numbers represent the number of times a gene was identified in each replication. Supplementary Table S8 shows the full list.

**Table 3.** Clone IDs Utilized in shRNA Knockdown Experiments

Clone ID	Gene Symbol	Gene ID	Ref. Sequence	Target Sequence
TRCN0000071724	*ATF4*	11911	NM_009716	CCAGAGCATTCCTTTAGTTTA
TRCN0000301646	*ATF4*	11911	NM_009716	CTAGGTCTCTTAGATGACTAT
TRCN0000288208	*BACH1*	12013	NM_007520	GCTCGACTGTATCCATGACAT
TRCN0000084279	*BACH1*	12013	NM_007520	GCGTACACAATATCGAGGAAT

**Figure 5.**
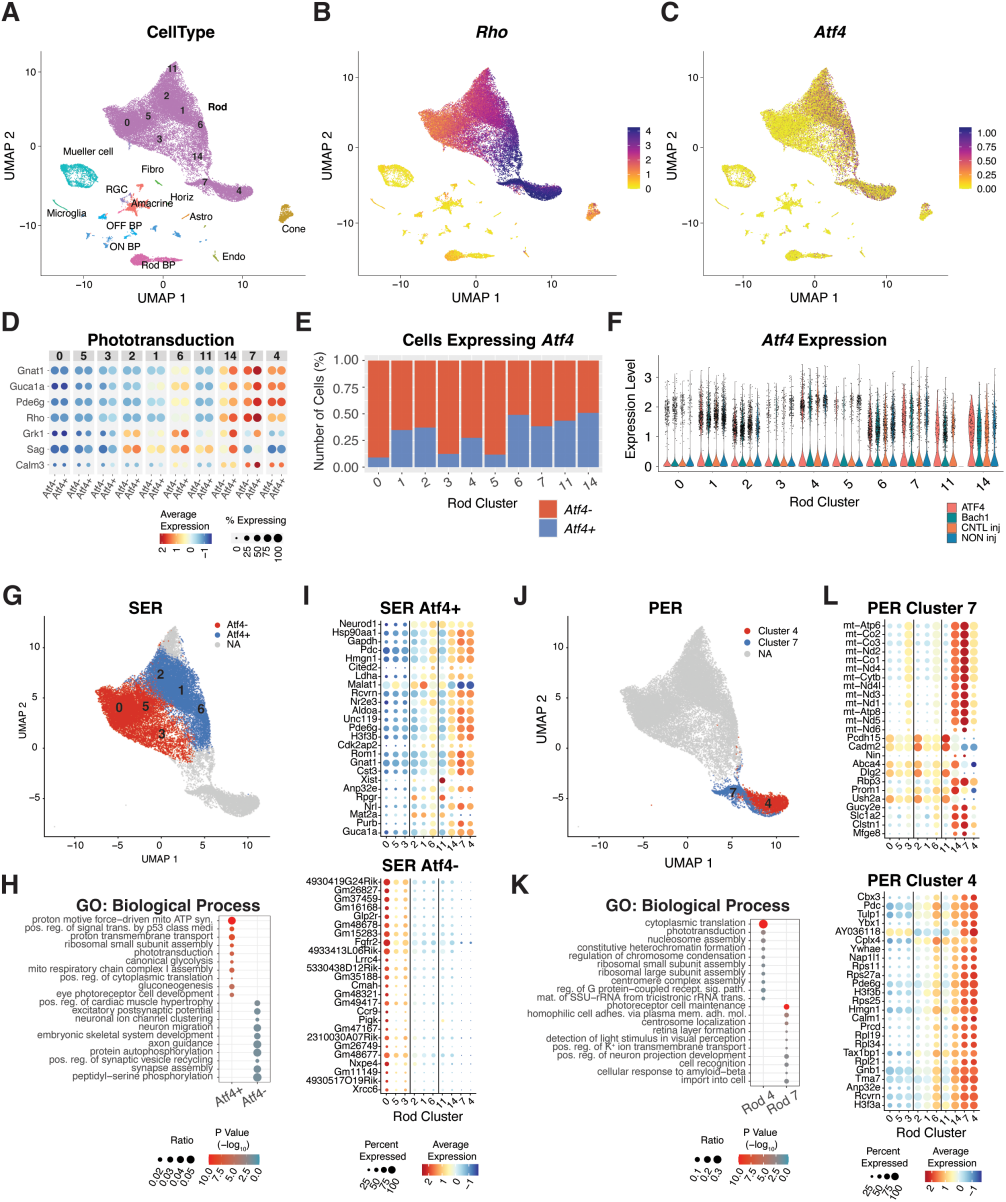
**scRNA-seq revealed that ATF4 downregulation is correlated with a decrease in NRL-mediated transcription.** (**A**) UMAP visualization of cell annotations with numbers identifying different rod clusters. (**B**) UMAP of *Rhodopsin* expression [log-normalized sctransform (SCT) defined as log1p value] indicating rod photoreceptor population. (**C**) UMAP of *Atf4* expression (log-normalized SCT value) across dataset. (**D**) Multi-dot plot depicting expression (*Z*-score of log-normalized SCT value) of phototransduction gene expression having both NRL and ATF4 binding at their promoter, separated by *Atf4^+^* versus *Atf4^–^* cells within rod clusters. (**E**) Stacked bar graph depicting the percentage of cells that were detected as positive for *Atf4* expression (*blue*) versus negative for *Atf4* expression (*red*) separated by rod cluster. (**F**) Multi-violin plot representing the expression level (log-normalized decontXcounts) of *Atf4* in each cluster broken down by sample type: *Atf4*-shRNA injected (*salmon*), *Bach1*-shRNA injected (*teal*), control-injected (*orange*), and non-injected (*blue*). (**G**) Cluster representation of SERs with high *Atf4* expression (*blue*) and low Atf4 expression (*red*). (**H**) Dot plot of top 10 enriched pathways based on genes differentially expressed between SER clusters with high *Atf4* expression (*Atf4^+^*) versus SER clusters with low *Atf4* expression (*Atf4^–^*). (**I**) Top 25 upregulated genes in *Atf4*-high SERs (*top*) and *Atf4*-low SERs (*bottom*). (**J**) Cluster representation of PERs with low *Atf4* representation (cluster 4, *red*) and high *Atf4* representation (cluster 7, *blue*). (**K**) Dot plot of enriched pathways based on genes differentially expressed between PER cluster 4 versus 7. (**L**) Top 25 differentially expressed genes from cluster 7 and cluster 4 (*bottom*). UMAP, uniform manifold approximation and projection; RGC, retinal ganglion cell; Horiz, horizontal cells; OFF BP, OFF cone bipolar cells; ON BP, ON cone bipolar cells; rod BP, rod bipolar cells; Astro, astrocytes; Endo, endothelial cells; GO, Gene Ontology; *P* value, Benjamini–Hochberg adjusted *P*.

Annotation of scRNA-seq data using known cell type markers resulted in 34 clusters representing the six major retinal neurons, glial and endothelial cells, and fibroblasts (Fig. 5A). Rod photoreceptors, identifiable by *Rho* expression (Fig. 5B), comprised 74.1% cells in the dataset. *Atf4* was present in most retinal cell types but was predominately detected in rods (Fig. 5C). We then assessed whether genes possessing an ATF4 binding site in the promoter are expressed at different levels in ATF^+^ versus ATF^–^ rods. We noted higher expression of phototransduction genes, such as *Rho*, *Gnat1*, and *Pde6g*, which contain CUT&Tag peaks of NRL and ATF4 (see Fig. 4B), in cells with high *Atf4* expression (Fig. 5D).

Despite not being able to confirm *Atf4* mRNA KD and provide more direct in vivo evidence, we leveraged the broad range of *Atf4* expression (Figs. 5E, 5F; Supplementary Fig. S4F, S4G) to indirectly infer its impact on rod gene transcription. We previously hypothesized a gradient of rod photoreceptors with efficient phototransduction profiles that appear along a continuum rather than a monolithic group.^48^ This continuum can be described with one type of rod photoreceptor being at each extreme: synaptic transmission-efficient rods (SERs), which can be differentiated at the RNA level from phototransduction-efficient rods (PERs) by relative expression levels of synaptic and phototransduction genes,^48^ such as Syntaxin 1 and Recoverin (Supplementary Fig. S4H), respectively. Thus, SERs and PERs may represent subpopulations of rods enriched for synaptic or phototransduction pathway machinery, respectively. One important nuance is that phototransduction genes are highly expressed at the PER end of the continuum and can dominate differential expression analyses, potentially obscuring subtler differences at the SER end. We therefore decided to divide the data into these two subsets and analyzed these separately rather than lumping all rods into a single comparison. Of the 10 rod clusters, SERs could be identified as clusters 0, 1, 2, 3, 5, and 6, whereas PERs were represented in clusters 4 and 7 based on expression of respective gene markers. Cluster 11 did not include any cells from the NON_Inj sample which may be a consequence of the subretinal injection procedure; therefore, we removed this cluster from downstream analysis. Cluster 14 was the smallest cluster, containing 1606 cells, and it showed mixed characteristics, therefore, it could reasonably be classified as either PER-like or SER-like (Supplementary Fig. S4H). To reduce ambiguity and avoid confounding effects driven by highly expressed phototransduction genes, we excluded this cluster from downstream analyses, as well. Though clusters 11 and 14 were excluded from further DE analysis, we included the respective expression plots for completeness.

We observed several SER clusters with relatively low populations of *Atf4*^+^ cells (clusters 0, 3, and 5; mean = 11%) when compared to the rest of the clusters, which had over 35% *Atf4*-expressing cells (Fig. 5E). Therefore, we compared similar SER cluster groupings with elevated populations of *Atf4*-expressing cells (clusters 1, 2, and 6) and those with low populations of *Atf4* expressing cells (clusters 0, 3, and 5) (Fig. 5G). Our analyses uncovered 745 genes that were significantly differentially expressed (DE); of these, 539 are upregulated in clusters showing the elevated population of *Atf4* cells (Supplementary Table S4). Functional gene enrichment (FGE) analysis of DE genes revealed several pathways, including ATP production, glycolysis, and phototransduction, that were significantly higher in *Atf4* high-population SER clusters. In contrast, *Atf4* low-population SER cells are enriched in more synaptic connection pathways (Fig. 5H; Supplementary Table S5). Furthermore, in *Atf4* high-population SERs, many rod genes—including *Nrl*, *Pdeg6*, *Nr2e3*, *Gnat1*, and *Rho*—exhibited significantly higher expression compared to *Atf4* low-population SER cells (Fig. 5I). We also observed that, although clusters 0, 3, and 5 contained fewer cells expressing *Atf4*, the expression of *Atf4* in those cells was significantly higher than in clusters 1, 2, and 6 (*P* < 1.0 × 10^−53^) (Supplementary Fig. S5F).

We next compared PER cluster 7 with cluster 4 (Fig. 5J) to assess whether *Atf*4-regulated expression differs in rods more specialized for phototransduction. We observed that clusters 4 and 7 possessed different expression levels of *Atf4* (Fig. 5F; Supplementary Fig. S4F, left panel), with cluster 4 showing higher and less variant expression. Of the 122 DE genes, 86 showed higher expression in cluster 7 (Supplementary Table S6). FGE analysis indicated enrichment of phototransduction or visual perception genes in both clusters 4 and 7 (Supplementary Table S7). The key difference is that cluster 4 was enriched in pathways associated with cytoplasmic translation and chromosome condensation (Figs. 5K, 5L), representing high transcriptionally active rods, whereas cluster 7 was enriched in mitochondrial genes along with photoreceptor cell maintenance and ion transport.

## Discussion

Cell-type-specific protein–protein interactions among transcription regulatory proteins occur in a spatially and temporally restricted manner to generate precision in gene expression patterns,^5^^,^^49^^,^^50^ including in the retina.^51^ NRL holds a central role in rod photoreceptor development and functional maintenance^20^^,^^30^^,^^52^ and is a viable target for therapies of many retinal diseases.^53^^–^^55^ To expand the specificity of NRL-mediated gene regulation in rods, we used both in vitro and in vivo systems to identify potential interactors and characterized their interactions in a physiologically relevant context. Across independent Y2H screens, ATF4 consistently emerged as the only bZIP TF that was robustly detected. Aside from NRL itself, BACH1 was the only other bZIP protein detected from co-immunoprecipitation experiments using adult retina. This is notable because c-Jun expression and its interaction with NRL are only detected in the developing retina, suggesting temporal or context dependence in functional interactions.^38^ Together, our findings highlight ATF4 as a strong and direct NRL-interacting partner and BACH1 as a modulator of NRL function, establishing a foundation for dissecting how specific bZIP heterodimers diversify their regulatory functions in the retinal photoreceptors.

Many TFs undergo homo- and/or heterodimerization to further control their DNA binding specificity, thereby enabling precise spatiotemporal control of transcription and orchestrating unique expression patterns in distinct cell types.^56^^,^^57^ At least 53 unique bZIP-containing proteins have been mapped in the human genome. Among these, CCAAT/enhancer binding protein (C/EBP), ATF4, ATF2, JUN, and the small MAF proteins are known to both homodimerize and heterodimerize with other bZIP proteins, whereas Fos, CNC, and large MAFs are primarily known for their promiscuous heterodimerization with other bZIP-containing proteins.^10^^,^^11^^,^^58^ Notably, ATF4 can heterodimerize with C/EBP, Fos, and nuclear factor erythroid 2-related factor 2 (NRF2).^11^^,^^59^ ATF4 is the first bZIP protein (reported here) that can heterodimerize with NRL in the mature rod photoreceptors. Why other rod-expressed bZIP proteins, such as C/EBP and JunD, were not detected in our screens remains an enigma. It is possible that interactions of NRL with other leucine zipper proteins are context dependent, transient, or, like BACH1, require additional factors. Additionally, the full-length NRL may be necessary to achieve a stable three-dimensional configuration of heterodimers in Y2H screens. Furthermore, specific post-translational modifications may be needed to facilitate these interactions and formation of NRL heterodimers.

Although ATF4 and BACH1 are both bZIP proteins, the two transcription factors appear to modulate NRL activity in opposite ways. We hypothesize that BACH1 likely functions as a negative regulator of NRL (based on luciferase reporter data) and is detected with NRL transiently or at fewer loci (reflected by PLA experiments) to fine-tune NRL-mediated transcription. BACH1–NRL interaction may be facilitated by other proteins within the transcription complex at select promoters. The direct Y2H assay supports this hypothesis of an indirect NRL–BACH1 association. Our data thus suggest that ATF4 and BACH1 modulate NRL-mediated gene expression through distinct mechanisms and in opposite directions.

The scRNA-seq data provided further insights into the protein interactions reported here. Although both clusters of PERs (clusters 4 and 7) were functionally enriched for processes involving light perception, mitochondrial genes were more differentially upregulated in cluster 7 compared to cluster 4. We observed that a subset of *Atf4*^+^ SERs exhibited higher expression of phototransduction genes, rendering them more similar to PERs, as indicated by their clustering. We therefore predict that rod photoreceptors across the retina may function with distinct phototransduction efficiency. We note that cells corresponding to SER clusters expressed higher levels of *Nrl* transcripts (clusters 1, 2, and 6) but had low *Atf4* expression; however, these clusters included a larger proportion of *Atf4*^+^ cells compared to SER clusters with lower *Nrl* expression (clusters 0, 3, and 5) and high *Atf4*. We hypothesize that ATF4 synergistically enhances NRL activity in cells where NRL expression is relatively low, allowing these rods to achieve higher transcriptional output despite reduced NRL levels.

Both ATF4 and BACH1 are established stress response proteins, which is especially notable for photoreceptors, given their exceptionally high metabolic demands and prolonged exposures to damaging light. ATF4 is involved in the integrated stress response, controlling endoplasmic reticulum stress and redox networks among others and working to restore metabolic homeostasis or leading to apoptosis when under chronic stress.^60^^,^^61^ Nonetheless, ATF4 function extends beyond stress response, as its binding to DNA is reported to prime gene promoters for enhanced activation.^62^ We suggest that this ability of ATF4 leads to augmented phototransduction gene expression in cooperation with NRL, which in turn reflects varying but specific stress responses for maintaining photoreceptor health and survival. Along these lines, we propose that rod-specific interactions of NRL with ATF4 and BACH1 might be helpful in protecting photoreceptors during oxidative stress and light damage via regulation of *Nrf2.*^59^^,^^63^^,^^64^ BACH1 is known to heterodimerize with small MAF family members to regulate CNC-sMaf binding element (CsMBE), and loss of BACH1–sMAF heterodimers is linked to derepression of *Hmox1* and subsequent neuronal degeneration.^65^ However, elevated BACH1 levels have also been associated with increased oxidative stress-induced DNA damage, which may explain the low levels of *Bach1* mRNA and BACH1 protein expression observed.^64^ This is the first reported interaction of the large MAF protein NRL with BACH1. It may be that NRL–BACH1 interaction is utilized to fine-tune responses to oxidative stress in rod photoreceptors. Together, our studies provide new insights into NRL function and indicate a potential role of ATF4 and BACH1 to rod-specific stress response through physiological interaction with NRL.

## Conclusions

Our studies implicate cell type–restricted TF interactions in establishing unique gene expression patterns in rod photoreceptors of the mammalian retina. Future investigations are needed to define the contributions of ATF4 and BACH1 to NRL and CRX-mediated photoreceptor homeostasis and to examine their roles under physiological and stress conditions.

## Supplementary Material

Supplement 1

Supplement 2
