# Supplementary material for: Stress Responsive bZIP Transcription Factors ATF4 and BACH1 Cooperate With MAF-Family bZIP Protein NRL to Fine-Tune Rod Photoreceptor Gene Expression
Source: Invest Ophthalmol Vis Sci. 2026 Jun 5;67(6):9. doi: 10.1167/iovs.67.6.9 (PMC13249099; doi:10.1167/iovs.67.6.9)
Supplement: Supplement 1 [file iovs-67-6-9_s001.pdf]

Supplementary Material

Stress responsive bZIP transcription factors ATF4 and BACH1 cooperate with Maf-family bZIP protein NRL to fine-tune gene expression in rod photoreceptors of the mammalian retina

Kiam Preston Jr.[1,2], Madhuri Arya PhD[1], Anjani Kumari PhD[1], Jacob Nellissery PhD[1], Matthew Brooks[1], Zachary Batz PhD[1], Xulong Liang PhD[1], Gianluca Tosini PhD[2], Anand Swaroop PhD[1*]

Supplemental Figure Legends

Fig. S1

(A) Co-immunoprecipitation from nuclear extract followed by western blot using NRL as probe in HEK293 cells. **(B)** Full plate images of Y2H screening performed on DDO, DDO/X/O, QDO/X/A.

Abbreviations: Basic Domain BD; Control: P-53 and Large T-antigen; Double dropout agar with X-Alpha-Gal and Aureobasidin A, DDO/X/O; Double dropout agar, DDO; Extended Homology Region, EHR; GBKT7 empty vector, BD; Immunoglobulin G, IgG; Immunoprecipitation, IP; Leucine Zipper, LZ; NRL leucine zipper domain, NRL LZ; pGADT7 empty vector, AD; Quadruple dropout agar with X-Alpha-Gal and Aureobasidin A, QDO/X/A; Yeast two-hybrid, Y2H.

Fig. S2

(A) Proximity ligation assay for DHX9 and NRL in HEK293 cells; Teal: DAPI nuclear stain; Magenta: PLA signal. **(B)** Proximity ligation assay for empty vector and Dhx9 in HEK293 cells; Teal: DAPI nuclear stain; Magenta: PLA signal. **(C)** Immunofluorescent co-staining of no primary control in mouse retina, Grey: DAPI, Teal/Magenta: Alexa 488, Red/Yellow: Alexa 555. **(D)** Immunofluorescent co-staining of DHX9 and NRL in mouse retina, Grey: DAPI, Teal/Magenta: DHX9, Red/Yellow: NRL. **(E)** Proximity ligation assay on WT mouse retina section for DHX9 and NRL, **(F)** DHX9 and NRL in an NRLKO mouse retina, **(G)** No primary control WT mouse retina, **(H)** No primary control NRLKO mouse retina, **(I)** ATF4 and NRL in an NRLKO mouse retina, **(J)** BACH1 and NRL in an NRLKO mouse retina; Blue: DAPI, Yellow: PLA signal.

Abbreviations: 4’,6-diamidino-2-phenylindole, DAPI; Inner nuclear layer, INL; Ganglion cell layer, GCL; NRL knockout, NRLKO; Outer nuclear layer, ONL; Stimulated Emission Depletion microscopy, STED.

Fig. S3

(A) Genome-wide distribution of BACH1, ATF4, and ATAC peaks. **(B)** Summary of overlaps between BACH1, ATF4, and ATAC peaks. **(C)** Histogram of average BACH1 (left) and ATF4 (right) read counts detected over accessible footprints identified by ATAC-seq.

Fig. S4

(A) Western blot of nuclear extract from HEK293 cells transfected with shRNA constructs against *Atf4* with appropriate empty vector and non-transfected control. **(B)** Western blot of nuclear extract from MEF cells transfected with shRNA constructs against *Bach1* with appropriate empty vector and non-transfected control.

Abbreviations: Human Embryonic Kidney Cells, HEK293; Mouse Embryonic Fibroblasts, MEF.

Fig. S5

(A) Single cell sequencing depth of each sample type. **(B)** UMI counts BArcode elbow plots for each sample type. **(C)** Number of genes per cell (upper) and UMI per cell (lower) with each sample type's median represented by a black line in the violin density plot. **(D)** UMAP of principal components 1 and 2 visualizing UMI per cell for all samples. **(E)** UMAP of cell types identified following clustering broken down by sample type. **(F)** Expression level of *Atf4* in rod cells expressing *Atf4* (left) and expression of *Bach1* in rod cells (right). **(G)** *Atf4* expression in rod clusters broken down by sample type. **(H)** *Syntaxin1* and *Recoverin* as a marker of SERs. **(I)** Expression of *Nrl* as a marker of PERs. Abbreviations: Phototransduction Efficient Rods, PERs; Synaptic Efficient Rods, SERs.

Fig. S1

A

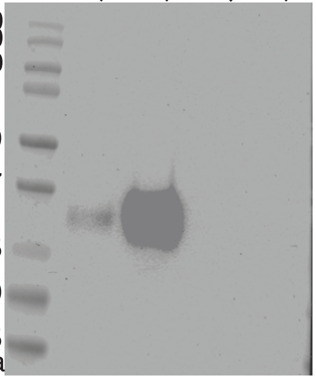

Western Blot: HEK293 Nuclear Extract

B

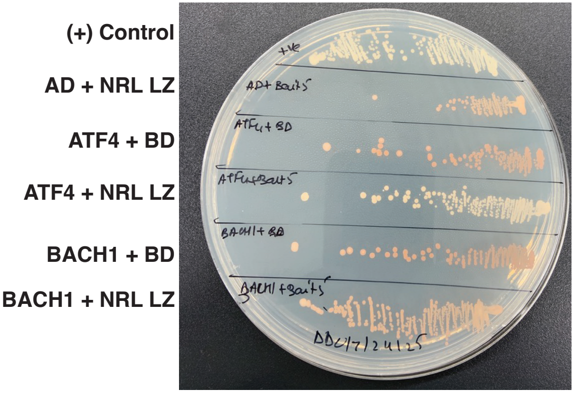

Y2H Double Dropout Agar

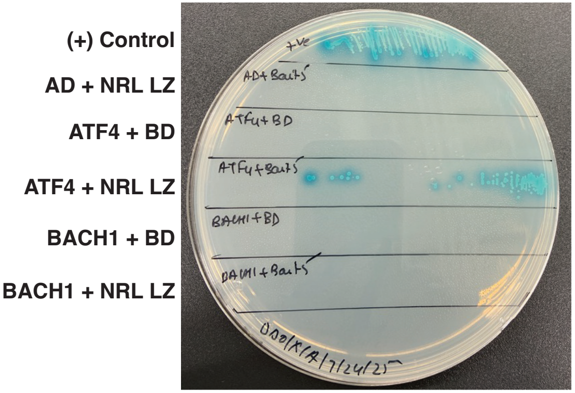

Y2H Double Dropout Agar with x-Alpha-Gal and Aureobasidin A

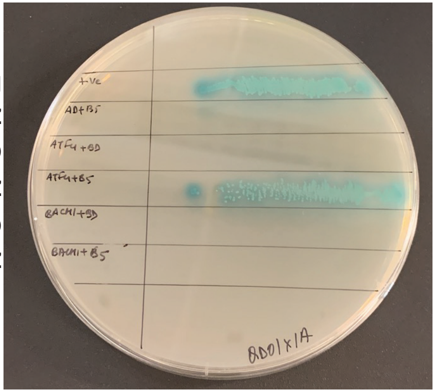

Y2H QuadrupleDropout Agar with x-Alpha-Gal and Aureobasidin A

Fig. S2

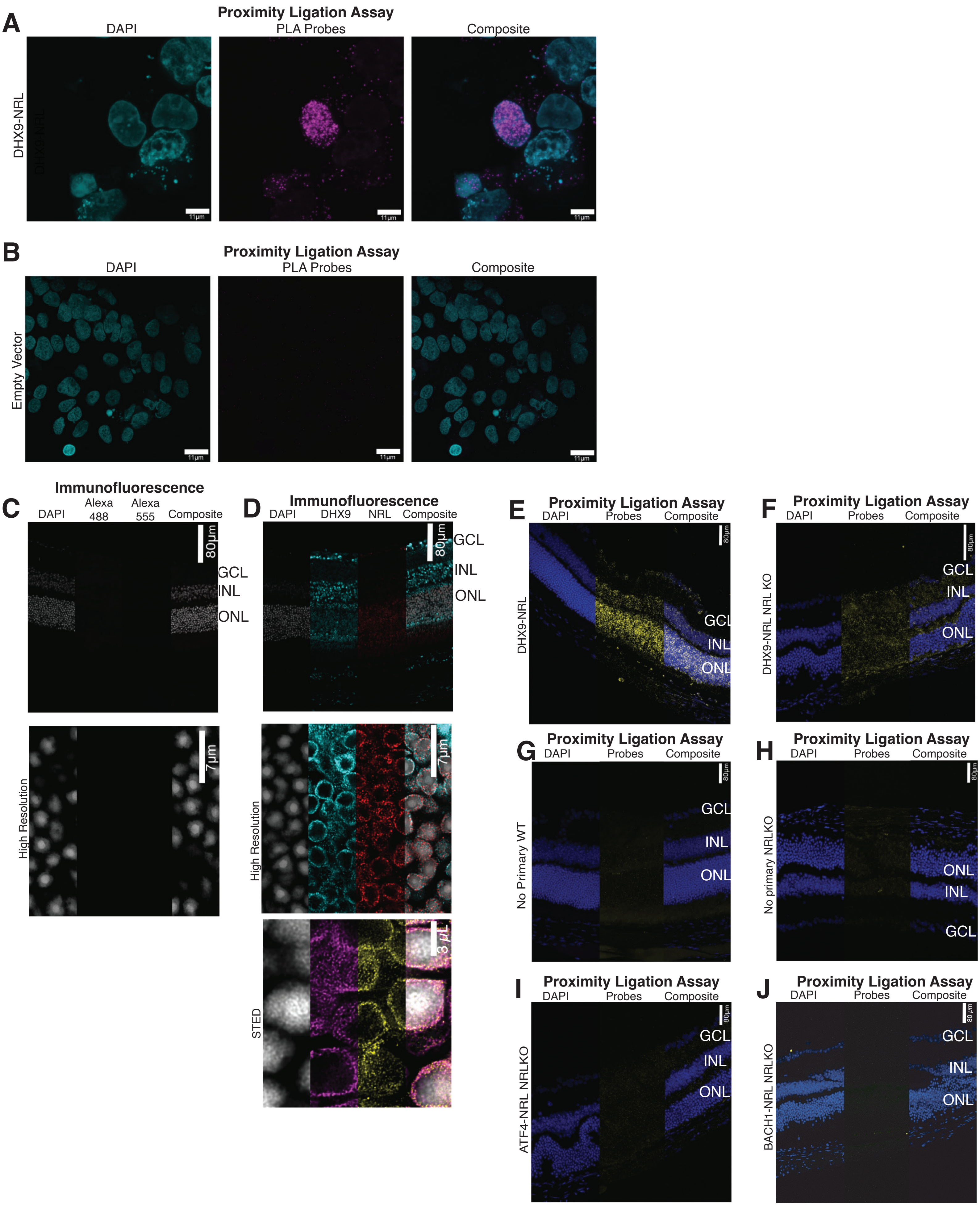

A
Proximity Ligation Assay
DAPI
PLA Probes
Composite
DHX9-NRL
11μm
B
Proximity Ligation Assay
DAPI
PLA Probes
Composite
Empty Vector
11μm
C
Immunofluorescence
DAPI
Alexa 488
Alexa 555
Composite
80μm
GCL
INL
ONL
High Resolution
7μm
D
Immunofluorescence
DAPI
DHX9
NRL
Composite
80μm
GCL
INL
ONL
High Resolution
7μm
STED
3 μm
E
Proximity Ligation Assay
DAPI
Probes
Composite
DHX9-NRL
GCL
INL
ONL
F
Proximity Ligation Assay
DAPI
Probes
Composite
DHX9-NRL NRL KO
GCL
INL
ONL
G
Proximity Ligation Assay
DAPI
Probes
Composite
No Primary WT
GCL
INL
ONL
H
Proximity Ligation Assay
DAPI
Probes
Composite
No primary NRLKO
ONL
INL
GCL
I
Proximity Ligation Assay
DAPI
Probes
Composite
ATF4-NRL NRLKO
GCL
INL
ONL
J
Proximity Ligation Assay
DAPI
Probes
Composite
BACH1-NRL NRLKO
GCL
INL
ONL

Fig. S3

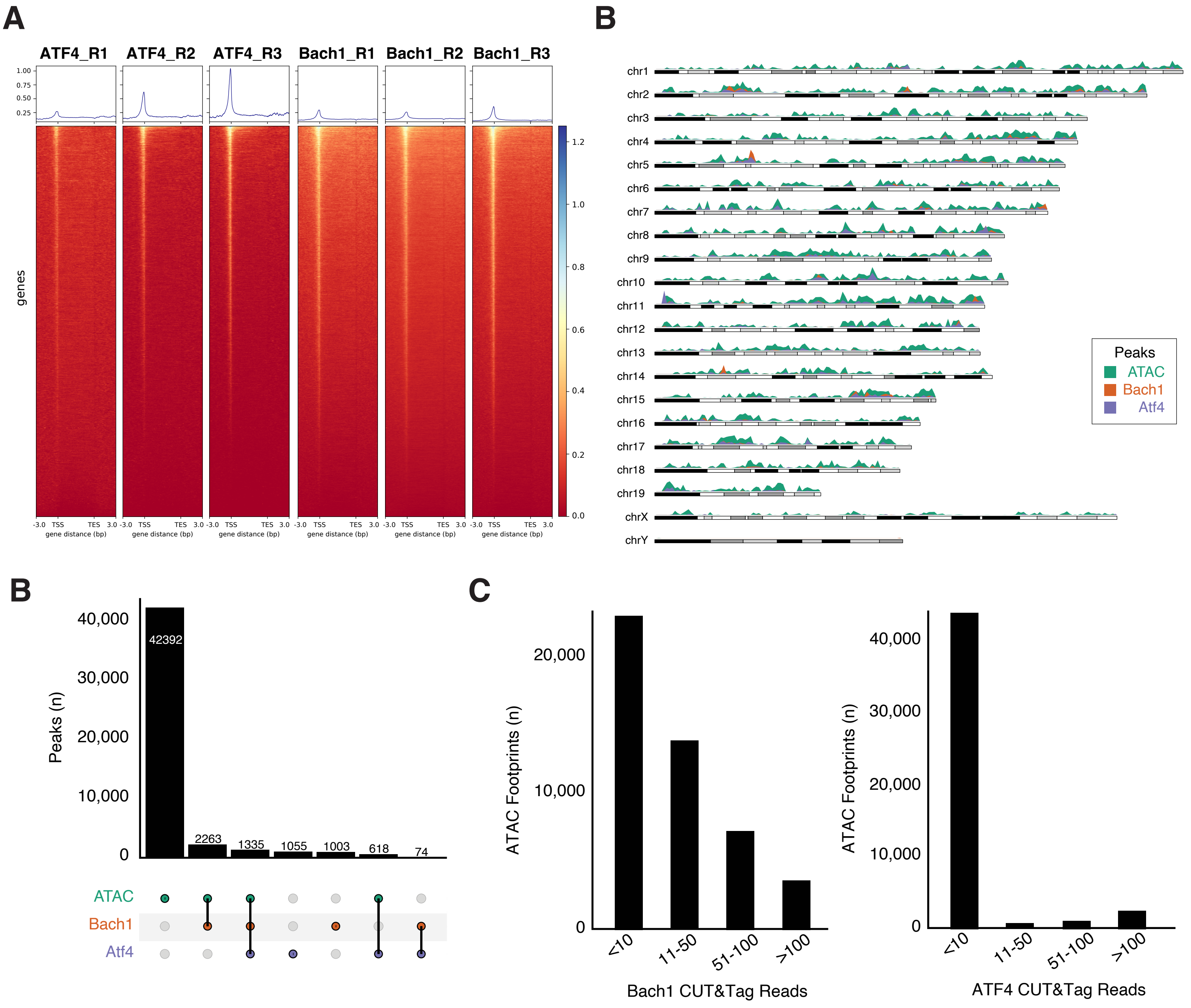
A
ATF4_R1
ATF4_R2
ATF4_R3
Bach1_R1
Bach1_R2
Bach1_R3
1.00
0.75
0.50
0.25
genes
1.2
1.0
0.8
0.6
0.4
0.2
0.0
-3.0 TSS TES 3.0
gene distance (bp)
B
chr1
chr2
chr3
chr4
chr5
chr6
chr7
chr8
chr9
chr10
chr11
chr12
chr13
chr14
chr15
chr16
chr17
chr18
chr19
chrX
chrY
Peaks
ATAC
Bach1
Atf4
B
Peaks (n)
40,000
30,000
20,000
10,000
0
42392
2263
1335
1055
1003
618
74
ATAC
Bach1
Atf4
C
ATAC Footprints (n)
20,000
10,000
0
<10
11-50
51-100
>100
Bach1 CUT&Tag Reads
ATAC Footprints (n)
40,000
30,000
20,000
10,000
0
<10
11-50
51-100
>100
ATF4 CUT&Tag Reads

Fig. S5

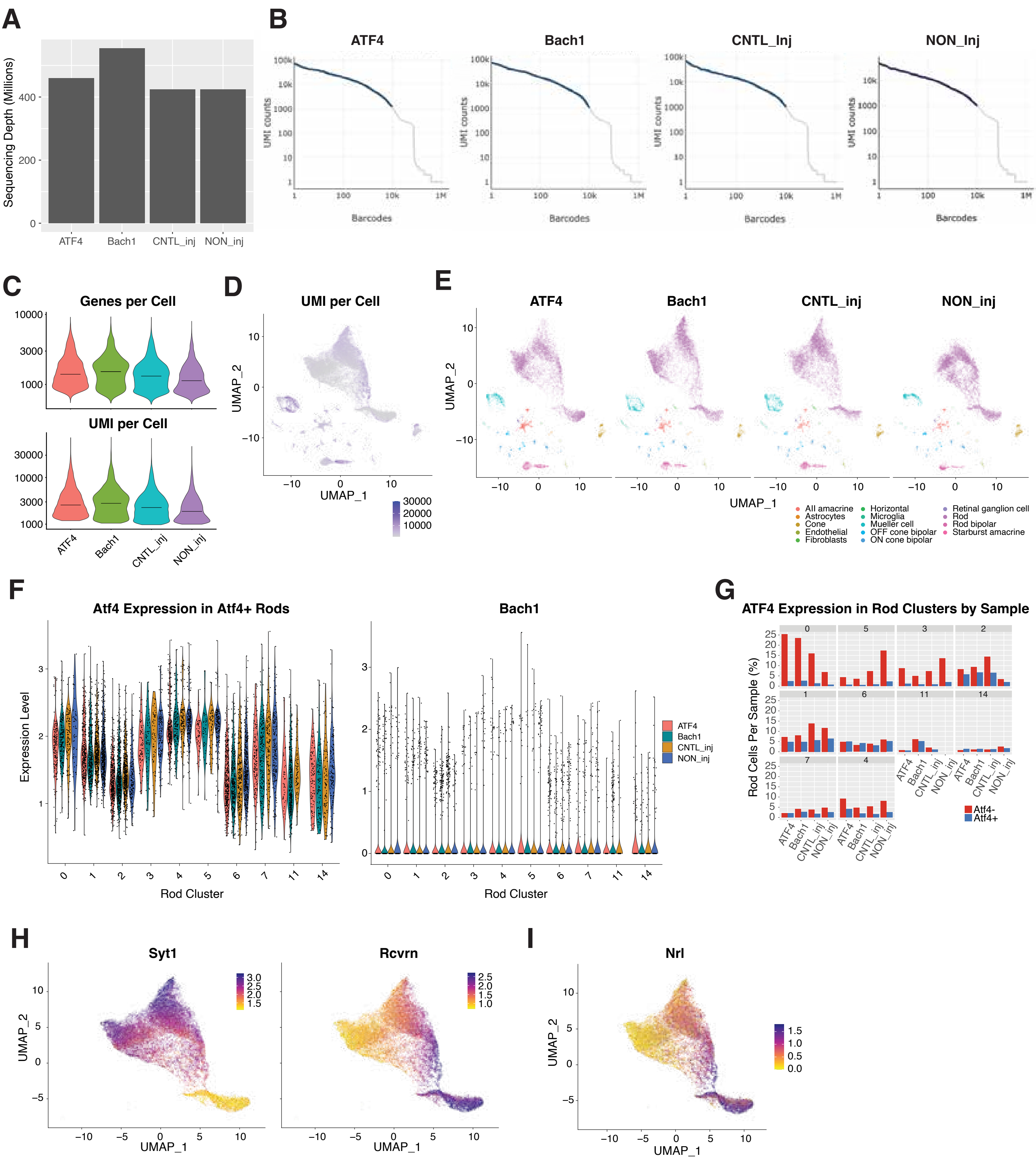

A
Sequencing Depth (Millions)
ATF4
Bach1
CNTL_inj
NON_inj
B
ATF4
Bach1
CNTL_Inj
NON_Inj
UMI counts
Barcodes
C
Genes per Cell
UMI per Cell
D
UMI per Cell
UMAP_1
UMAP_2
E
ATF4
Bach1
CNTL_inj
NON_inj
All amacrine
Astrocytes
Cone
Endothelial
Fibroblasts
Horizontal
Microglia
Mueller cell
OFF cone bipolar
ON cone bipolar
Retinal ganglion cell
Rod
Rod bipolar
Starburst amacrine
F
Atf4 Expression in Atf4+ Rods
Bach1
Expression Level
Rod Cluster
G
ATF4 Expression in Rod Clusters by Sample
Rod Cells Per Sample (%)
Atf4-
Atf4+
H
Syt1
Rcvrn
I
Nrl

Fig. S4

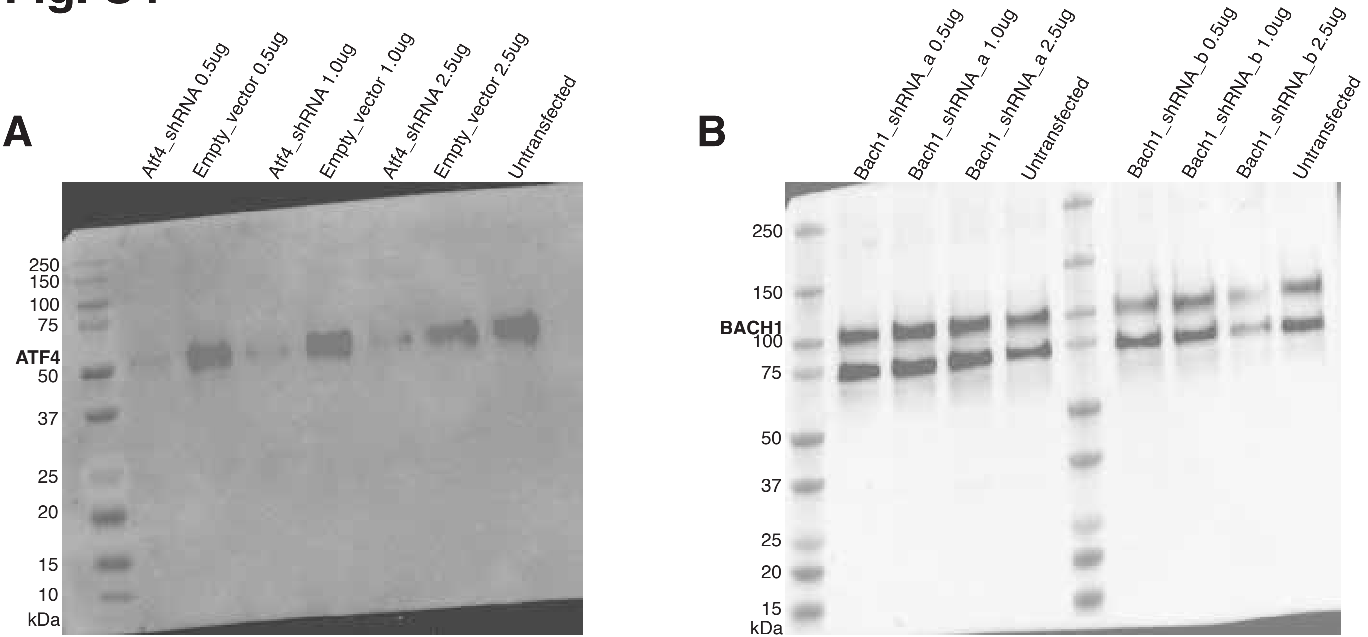

Western Blot: HEK293 Nuclear Extract shRNA Transfection

Western Blot: MEF Nuclear Extract shRNA Transfection

Supplemental Table Legends

Table S1

Genelist from BACH1 CUT&Tag, identifying genomic locations where BACH1 is bound to protein coding gene promoters.

Table S2-S3

FGE results from CUT&Tag gene lists related to Fig. 4. FGE of Gene Ontology Biological Processes for genes with ATF4 or BACH1 peaks located in the promoter regions. GeneRatio indicates the number of genes identified in the pathway divided by the total number of genes. BgRatio represents the number of genes in the ontology term divided by the total number of genes in the dataset. The 'p.adjust' column indicates the Benjamini-Hochberg false discovery rate.

Table S4-S5

scRNA-seq differential expression analysis results related to Fig. 5. Results are from analysis of genes from shRNA Transfected, or control samples for P21-P23 day old CD1 mouse retina. Table S4 contains results from FGE comparing rod clusters 0, 3 and 5 with rod clusters 1, 2 and 6. P-val-adj indicates the Benjamini-Hochberg false discovery rate. Table S5 contains Gene Ontology of Biological Processes for genes differentially expressed in these comparisons. GeneRatio indicates the number of genes identified in the pathway divided by the total number of genes. BgRatio represents the number of genes in the ontology term divided by the total number of genes in the dataset. The 'p.adjust' column indicates the Benjamini-Hochberg false discovery rate.

Table S6-S7

scRNA-seq differential expression analysis results related to Fig. 5. Results are from analysis of genes from shRNA Transfected, or control samples for P21-P23 day old CD1 mouse retina. Table S6 contains results from FGE comparing rod cluster 4 with rod clusters 7. P-val-adj indicates the Benjamini-Hochberg false discovery rate. Table S7 contains Gene Ontology of Biological Processes for genes differentially expressed in these comparisons. GeneRatio indicates the number of genes identified in the pathway divided by the total number of genes. BgRatio represents the number of genes in the ontology term divided by the total number of genes in the dataset. The ‘p.adjust’ column indicates the Benjamini-Hochberg false discovery rate.
